# RhierBAPS: An R implementation of the population clustering algorithm hierBAPS

**DOI:** 10.12688/wellcomeopenres.14694.1

**Published:** 2018-07-30

**Authors:** Gerry Tonkin-Hill, John A. Lees, Stephen D. Bentley, Simon D.W. Frost, Jukka Corander

**Affiliations:** 1 Parasites and Microbes, Wellcome Trust Sanger Institute, Hinxton, Cambridgeshire, CB10 1SA, UK; 2 Department of Microbiology, New York University School of Medicine, New York, NY, 10016, USA; 3 The Alan Turing Institute, London, NW1 2DB, UK; 4 Department of Veterinary Medicine, University of Cambridge, Cambridge, Cambridgeshire, CB3 0ES, UK; 5 Department of Biostatistics, University of Oslo, Blindern, 0317, Norway; 6 Department of Mathematics and Statistics, University of Helsinki, Helsinki, 00014, Finland

**Keywords:** clustering, population structure, R

## Abstract

Identifying structure in collections of sequence data sets remains a common problem in genomics. hierBAPS, a popular algorithm for identifying population structure in haploid genomes, has previously only been available as a MATLAB binary. We provide an R implementation which is both easier to install and use, automating the entire pipeline. Additionally, we allow for the use of multiple processors, improve on the default settings of the algorithm, and provide an interface with the ggtree library to enable informative illustration of the clustering results. Our aim is that this package aids in the understanding and dissemination of the method, as well as enhancing the reproducibility of population structure analyses.

## Introduction

Identifying sub-populations in collections of genetic sequences is a common problem in population genetics, molecular ecology, epidemiology and microbiology. In general, the aim of genetic clustering algorithms is to identify separate panmictic clusters within a broader, more heterogeneous population. In large sequence data sets, it is helpful to identify smaller subpopulations which can be further analysed for associations with particular phenotypes as well as recombination ^1, 2^, as long as potential biases introduced through taking clusters from a larger population are taken into account ^3^.

A frequently used model assumes that each individual sequence is drawn from one of *K* distinct subpopulations with each cluster having its own set of allele frequencies. The aim is then to identify which cluster each sequence originates from and the corresponding allele frequencies within that cluster.

There are a number of methods for solving this problem including STRUCTURE ^4, 5^, snapclust ^6^ and BAPS (Bayesian Analysis of Population Structure ^7– 10^). The BAPS algorithm ^9, 11^ is distinct in that it attempts to estimate the partition of individual sequences into clusters directly by analytically integrating over the allele frequencies parameters for each subpopulation. This allows for the latent number of underlying subpopulations, *K*, to be estimated as part of the model fitting procedure. The hierBAPS algorithm extends this approach by enabling the investigation of a population at multiple resolutions. This is achieved by initially clustering the entire dataset using the BAPS algorithm before iteratively applying the algorithm to each of the resulting clusters.

Similar to other approaches ^4^, BAPS assumes that alleles are drawn independently from a multinomial distribution with a Dirichlet prior. However, unlike STRUCTURE, which uses Gibbs sampling to estimate the posterior distribution, BAPS attempts to find the partition of the data *S* that maximises the posterior probability of an allocation over all other possible allocations. A partition *S* is defined as the allocation of each sequence to one of *K* possible clusters. The maximum possible value of *K* is given in the hierBAPS algorithm. Here ** indicates the set of all possible partitions with up to *K*_max_ clusters. The hierBAPS algorithm attempts to choose *S* to maximise

$$P\left(S\text{|data}\right)=\frac{P\left(\text{data|}S\right)P\left(S\right)}{\Sigma_{S\in }P\left(\text{data|}S\right)P\left(S\right)}$$

where *P*(data *|S*) is the marginal likelihood of having the allele frequency parameters analytically integrated out leading to

$$P\left(\text{data|}S\right)=\prod_{i=1}^{K}\prod_{j=1}^{N_{l}}\left(\frac{\Gamma (\sum_{l}\;\alpha_{i\;jl})}{\Gamma (\sum_{l}\;\alpha_{i\;jl}\;+\;n_{i\;jl}\;)}\;\;\prod_{l=1}^{N_{A\left(j\right)}}\;\frac{\Gamma (\alpha_{i\;jl}\;+\;\;n_{i\;jl}\;)}{\Gamma (\alpha_{i\;jl})}\right)$$

where *n _i jl_* is the count for allele *l* at locus *j* in cluster *i* and *α _i jl_* is the corresponding hyper-parameter for the Dirichlet prior. The BAPS algorithm attempts to find the partition *S* that maximizes the posterior probability using a greedy stochastic search approach. A discretised uniform distribution of the cluster size *K* ( *K* = 1,…, *K*_max_) is used in hierBAPS to provide the prior probability of each partition *P*( *S*). The Dirichlet hyperparameters are set at $\frac{1}{N_{A\left(j\right)}}$ where *N*_A( *j*)_ is the number of of distinct alleles at locus *j*.

Currently, hierBAPS is only available as a MATLAB binary, which can be both difficult to install and use as separate runtime libraries are generally needed for different OS versions for MacOS X, Windows and Linux systems. The documentation is also lacking, making it difficult for less computationally experienced researchers to use. There is currently no clear guide on how to use the output of the MATLAB binary to produce informative plots for interpretation. Whilst there are other algorithms available to cluster genetic data in R, such as snapclust ^6^ and DAPC ^12^, neither make use of the partition approach used in BAPS. By providing an R implementation of hierBAPS, we aim to increase its usability and the reproducibility of analyses using the software.

## Methods

### Implementation

RhierBAPS is implemented in the R language ^13^. The core program relies upon the R packages ape ^14^, dplyr, gmp, purrr and ggplot2. Additional plotting functionality makes use of ggtree ^15^ and phytools ^16^. The structure of the code is very similar to the original MATLAB code and has similar runtimes. The development version of the package can be installed using devtools.

install.packages("devtools")
devtools::install_github("gtonkinhill/rhierbaps")

Unlike the MATLAB version, rhierBAPS by default only considers SNP loci that have a minor allele in at least two sequences. This has been found to improve the results of the analysis as although singleton SNPs are important when constructing phylogenies they introduce noise into the model used in hierBAPS leading to poorer quality clusterings. It is currently recommended that singletons SNPs are removed before running the MATLAB version of the software.

### Operation

RhierBAPS can be installed on any computer where R versions 3.5 and above can be installed. The package can be run using just a few lines of R code where the variable "fasta.file.name" should be replaced with the location of the FASTA formated multiple sequence alignment of the sequences of interest.

library(rhierbaps)

fasta.file.name <− system.file("extdata", "seqs.fa", package = "rhierbaps")
snp.matrix <−load_fasta(fasta.file.name)
hb.results <−hierBAPS (snp.matrix, max.depth = 2,  n.pops = 20, quiet = TRUE)

## Use cases

RhierBAPS requires a multiple sequence alignment in FASTA format. In all examples we make use of sequences from the *Bacillus cereus* Multi Locus Sequence Typing website ( https://pubmlst.org/bcereus/) ^17^. The sequences used are included as part of the R package.

The algorithm requires an initial number of clusters to be set which should be higher than the maximum number of expected clusters in the dataset. If a dataset is likely to contain many distinct lineages, for example, if there are many samples from many locations, then a higher initial number of clusters should be set. Conversely, if the samples are from only a small number of sites and little variation is expected then a smaller initial cluster size can be set. To get an idea of a good initial cluster size, agglomerative clustering with complete linkage using pairwise SNP distances can be performed initially. The number of levels over which clustering should be performed is also required as input to the algorithm.

In the preceding example, we ran rhierBAPS with 20 initial clusters at two clustering levels. Additional parameters that can be set include the number of cores to use and whether the program should generate progress information. The hierBAPS function generates a data frame indicating the assignment of sequences to clusters at each level. This, along with the marginal log likelihoods can be saved to file.

write.csv(hb.results$partition.df, file = "hierbaps_partition.csv", col.names = TRUE,
     row.names = FALSE)

save_lml_logs(hb.results, "hierbaps_logML.txt")

**Figure 1.**
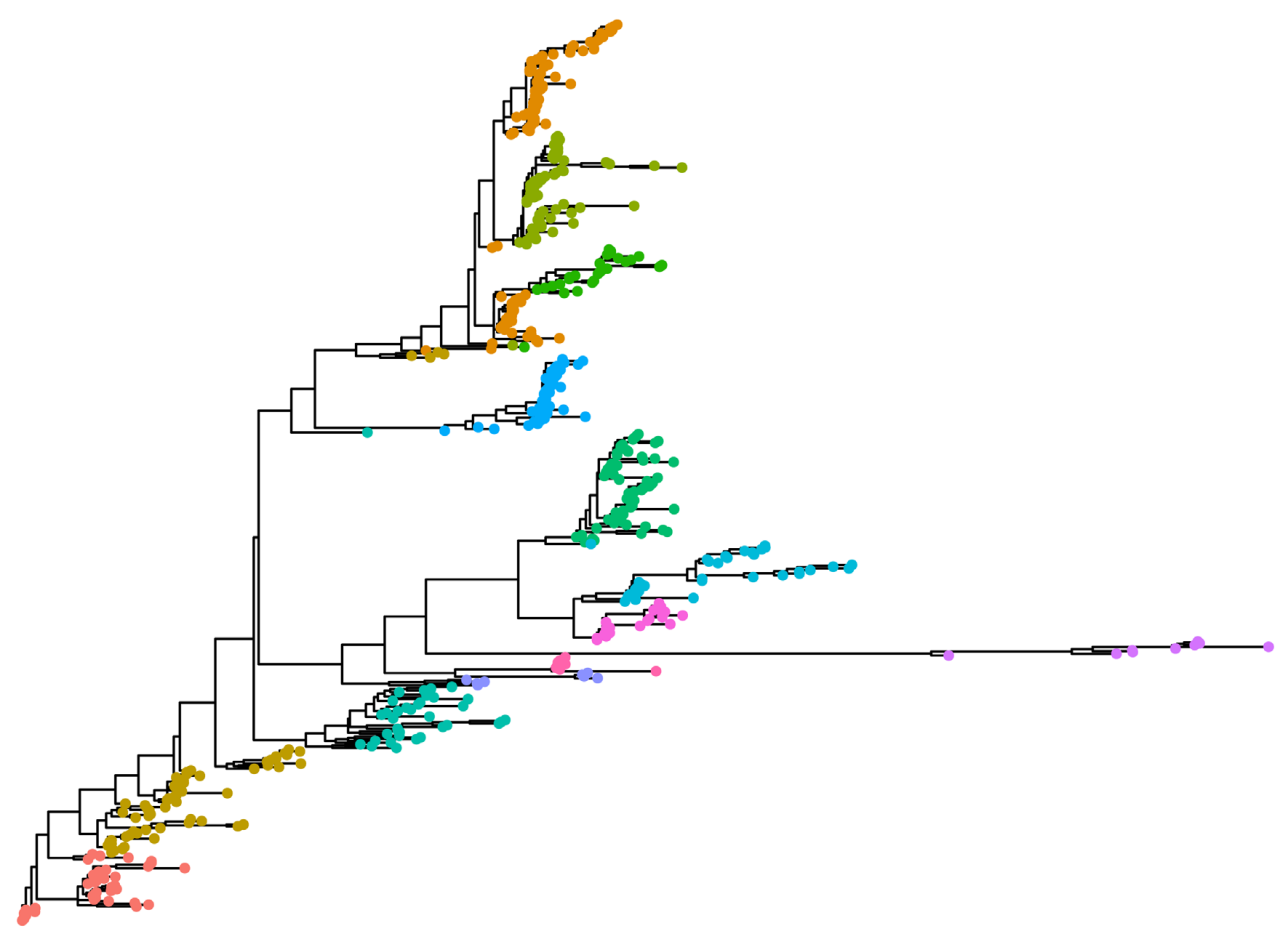
Phylogenetic tree built using Iqtree and annotated with the top level clusters identified using rhierBAPS.

Finally, as the program is written in R we are able to take advantage of the excellent plotting capabilities available. Given a phylogenetic tree generated using IQTREE ^18^ with model selection ^19^ using the command iqtree *−*s, we can annotate it with the BAPS clusters using ggtree ^15^ ( Figure 1).

newick.file.name <— system.file("extdata", "seqs.fa.treefile", package = "rhierbaps")
iqtree <— phytools::read.newick(newick.file.name)

gg <—ggtree(iqtree,layout="circular")
gg <—gg%<+%hb.results$partition.df
gg <—gg+geom_tippoint(aes(color=factor(‘level 1‘)))
gg

**Figure 2.**
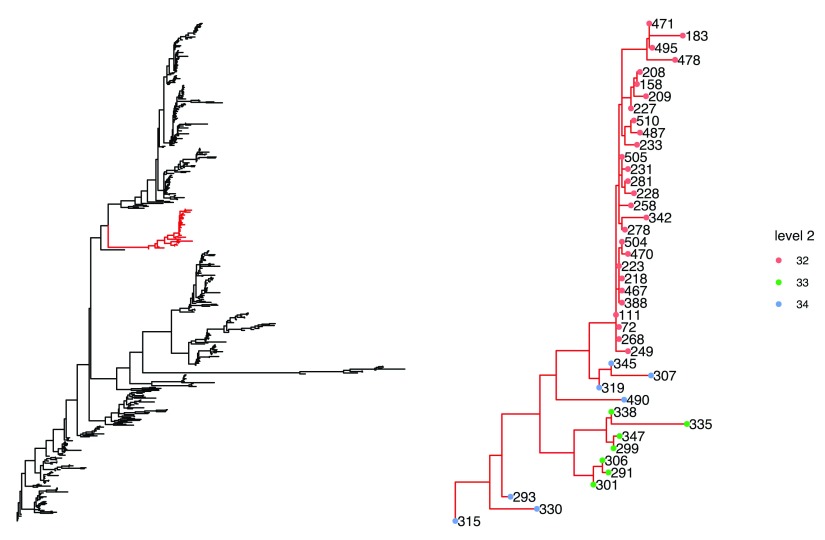
Phylogenetic tree focusing on the 9th cluster at the top level identified using rhierBAPS and plotted using the plot_sub_cluster function. The subsequent clustering at the 2nd level is indicated in the sub-tree to the right.

Additionally, the plot_sub_cluster function allows for the user to focus on one higher level cluster and investigate the sub cluster present within it. Here we investigate cluster 9 (highlighted in red), at the top most level ( Figure 2).

plot_sub_cluster(hb.results, iqtree, level = 1, sub.cluster = 9)

## Summary

Clustering is an essential component of many genetic analysis pipelines. We have presented rhierBAPS, an R package that implements the hierBAPS algorithm for clustering genetic sequence data. It is both easy to install and use, whilst providing additional plotting capabilities and the ability to run using multiple cores. We believe it will aid in the reproducibility of population structure analysis.

## Software availability

The package is available on CRAN: https://cran.r-project.org/web/packages/rhierbaps/index.html

Source code available from: https://github.com/gtonkinhill/rhierbaps

Archived source code as at time of publication: http://doi.org/10.5281/zenodo.1318958 ^20^

License: MIT
