# Supplementary material for: RhierBAPS: An R implementation of the population clustering algorithm hierBAPS
Source: Wellcome Open Res. 2018 Jul 30;3:93. [Version 1] doi: 10.12688/wellcomeopenres.14694.1 (PMC6178908; doi:10.12688/wellcomeopenres.14694.1)
Supplement: Peer review history

# Peer review: Referee response for version 1

**DOI:** 10.21956/wellcomeopenres.16000.r33842

**Published:** 2018-10-05

**Authors:** Sebastian Duchene

**Affiliations:** 1 Department of Biochemistry and Molecular Biology, University of Melbourne, Parkville, Vic, Australia

The authors report an implementation of hierBAPS for R, RhierBAPS, for determining the optimal number of clusters in population DNA sequence data. The program does not extend the methods in hierBAPS, but I appreciate that R is the default language in many bioinformatics pipelines.

 I have a few suggestions:

 - The program is easy to use, but I think that it is worth including a few data sets that can are readily available when the package is loaded into R. This would make the examples easier to run and understand.

 - I think that it would be valuable to see some results of choosing different minimum values of K to illustrate the sensitivity of the clustering to this parameter.

I have read this submission. I believe that I have an appropriate level of expertise to confirm that it is of an acceptable scientific standard.

# Peer review: Referee response for version 1

**DOI:** 10.21956/wellcomeopenres.16000.r33752

**Published:** 2018-09-17

**Authors:** Emmanuel Paradis

**Affiliations:** 1 ISEM,, University of Montpellier, CNRS, EPHE, IRD, Montpellier, France

The identification of groups (or clusters) with genetic or genomic data is one the basic questions asked by population geneticists. Several methods exist with various software implementations. The software described in this note is a valuable addition to the set of R packages for population genetics and related fields. I greatly appreciate to have this method implemented in R. The analytical integration is a really great feature and that makes this method attractive.

 I tried the package and the examples run very smoothly as expected. I also tried the main function with a small DNA alignment (the 'woodmouse' data in ape) and the results made sense. The graphical tools provided with the package are helpful, and the results output as a list in R make easy to use a custom graphical display. For instance, I was able to make my own display with functions in ape in just four lines of code.

 I have a few comments or suggestions that the Authors may find useful for future versions of their article and/or package.

 At present, it seems that the package handles SNP data. Does this mean that only biallelic DNA loci can be analysed? Can other types of biallelic genetic data be handled? What if more than two alleles are observed at a DNA site?

 One suggestion for future developments would be to better integrate with other data classes, particularly from ape and adegenet which are more and more widely used. Also, ape has now efficient links with the data classes used in BioConductor, which makes possible to integrate a wide range of approaches (bioinformatics, genomics, phylogenetics, population genetics) within R.

 It seems that the present package does not calculate the individual relative probabilities of assignment to the different clusters as done in Corander *et al*. ^1^. This might be a valuable addition for future versions, and it would help to compare the results from different methods, for instance using the nice compoplot function in adegenet.

I have read this submission. I believe that I have an appropriate level of expertise to confirm that it is of an acceptable scientific standard.

## References

[1] CoranderJMarttinenPSirénJTangJ: Enhanced Bayesian modelling in BAPS software for learning genetic structures of populations. *BMC Bioinformatics*.2008;9: 10.1186/1471-2105-9-539 539 10.1186/1471-2105-9-539 19087322PMC2629778
